# Legumes and pulses - a scoping review for Nordic Nutrition Recommendations 2023

**DOI:** 10.29219/fnr.v68.10484

**Published:** 2024-03-26

**Authors:** Liv Elin Torheim, Lars T. Fadnes

**Affiliations:** 1 Department of Physical Health and Ageing, Norwegian Institute of Public Health, Oslo, Norway; 2 Department of Global Public Health and Primary Care, University of Bergen, Bergen, Norway

**Keywords:** pulses, legumes, Fabaceae, dietary recommendations

## Abstract

Consumption of legumes and pulses is associated with various health outcomes. Therefore, when updating the Nordic Nutrition Recommendations (NNR), summarizing the best available evidence on key health outcomes regarded as relevant for the Nordic and Baltics related to the consumption of legumes was essential. The aim of this scoping review was to evaluate the updated evidence on the effect of the consumption of legumes and pulses on various health outcomes, as well as their dose-response relationship in updated systematic reviews and meta-analyses. The scoping review is built on a *de novo* systematic review published in 2023 and additional searches on the consumption of legumes and pulses and its various health outcomes, including cardiovascular disease (CVD), cancer, type 2 diabetes, and obesity. Current available evidence shows that the consumption of legumes and pulses is associated with a lower risk of several cancers (evidence: low-moderate), and lower all-cause mortality (evidence: moderate). The associations with CVDs are neutral or inverse, with studies generally showing favourable changes in biomarkers for CVDs. Legume consumption is associated with a lower risk of obesity (evidence: low). For type 2 diabetes, no association was found with incidence, but trials on consumption of legumes and pulses and biomarkers generally indicated protective effects. Overall, the current evidence supports dietary recommendations to increase the consumption of legumes and pulses.

## Popular scientific summary

- Legumes and pulses are good and sustainable sources of plant proteins, complex carbohydrates, fibre, and several micronutrients, and are generally low in fat and saturated fatty acids.
- The consumption of legumes and pulses is relatively low in the Nordic and Baltic countries.
- The effect of legumes and pulses intake on cardiovascular disease is unclear and limited due to low consumption among the studied populations.
- High intake of legumes and pulses has shown beneficial effects on cardiometabolic risk factors.
- Intake of legumes and pulses, and soy in particular, has been associated with lower risk of cancer and total mortality, but the certainty of the evidence is limited.

Legumes is a collective term for plants under the Fabaceae botanical family, and include various types of beans, lentils, peas, and soybeans (1). Pulses refer to the dry, edible seeds from legume plants, and include species such as beans, lentils, and peas (2). Fresh beans and peas as well as soy products are classified under legumes, but not necessarily under pulses. Peanuts classify botanically as legumes, but are usually classified as nuts in nutritional research in line with their culinary definition (3). In this scoping review, we use the term legumes referring to lentils, peas, and beans including soybeans. Legumes are good sources of proteins, complex carbohydrates, and fibre, and are generally low in fat and saturated fatty acids. Legumes contain between 17 and 40% protein (dry matter). Soybean has a high protein content (37%), and the protein has high digestibility and a complete protein quality containing all indispensable amino acids (4, 5). Legumes are rich in lysine; however, most are limited in one or more indispensable amino acids, typically the sulphur-containing amino acids, methionine and cysteine. Grains have a complementary amino acid profile to legumes, being rich in methionine and cysteine but low in lysine. Thus, consuming a varied diet with both legumes and cereals will for most ensure an adequate protein intake. The content of micronutrients differs between varieties, but several legumes are rich in folate, thiamine, potassium, magnesium, iron, and zinc, as well as bioactive compounds such as phytochemicals (1, 6). Legumes, and especially pulses, may contain anti-nutritional factors that can interfere with the absorption of nutrients and reduce the bioavailability of certain minerals. Anti-nutritional factors, such as phytates, trypsin inhibitors and tannins, can be reduced through soaking, cooking and fermentation (4).

Legumes form an important part of the food culture in many countries, but in many settings, including the Nordic and Baltic countries, the consumption is relatively low (7). Legumes are increasingly used as an alternative to meat, being a nutritious food with high protein content and a low environmental footprint (8). Protein concentrates and isolates from legumes are increasingly being used in meat analogues (9). These products may contain less fibres and have a high level of salt, and thus not necessarily have the same healthy nutritional profile as legumes that have been lightly processed through, for example, cooking. The protein digestibility of plant-based products can also change with processing (9). There has been an expansion in research on the health effects of legumes over the past years. The Nordic Nutrition Recommendations (NNR) from 2012 did not give recommendations for legumes, but legumes were mentioned several times. The Nordic countries have, to a varying degree, included legumes in their national dietary guidelines. The Norwegian dietary guidelines based on NNR2012 do not include legumes (10), whereas Sweden and Finland encourage the consumption of legumes, and Denmark, Estonia, Iceland, and Latvia give specific recommendations in amounts (7).

The objective of this scoping review is to transparently report the updated evidence on the consumption of legumes and associated health outcomes regarded as relevant for the Nordic and Baltic countries, as well as dose-response relationship between legumes and various health outcomes presented in updated systematic reviews and meta-analyses (Box 1).

*Box 1.* Background papers for Nordic Nutrition Recommendations 2023

- This paper is one of many scoping reviews commissioned as part of the Nordic Nutrition Recommendations 2023 (NNR2023) project (13).
- The papers are included in the extended NNR2023 report but, for transparency, these scoping reviews are also published in Food & Nutrition Research.
- The scoping reviews have been peer reviewed by independent experts in the research field according to the standard procedures of the journal.
- The scoping reviews have also been subjected to public consultations (see report to be published by the NNR2023 project).
- The NNR2023 committee has served as the editorial board.
- While these papers are a main fundament, the NNR2023 committee has the sole responsibility for setting dietary reference values in the NNR2023 project.

## Methods

Literature searches were screened to extract relevant evidence. No relevant, independent systematic reviews by multidisciplinary experts commissioned by national food or health authorities or international food and health organisations were identified by the NNR2023 Committee (11). However, a *de novo* systematic review for NNR2023 was conducted (3). The *de novo* systematic review screened 10,771 articles from MEDLINE, Embase, Cochrane, and Scopus. In addition, nine records were identified through screening articles in other systematic reviews on the topic. The review included 47 papers of which 42 were included in the meta-analyses on 25 unique cohorts with a total of 2,081,423 participants, and 14 randomized controlled trials with a total of 715 participants. Health outcomes presented were cardiovascular disease (CVD) and type 2 diabetes, and their associated biomarkers. Associations for high versus low consumption, per serving, and dose-response relationships were presented.

In addition to the *de novo* systematic review, we conducted additional searches for systematic reviews and meta-analyses on the health outcomes cancer, obesity, dementia, and all-cause mortality. We used a culinary definition of legumes including peas, lentils, and beans (but excluding coffee and cacao beans). Peanuts are not covered as they are culinarily considered as a nut. MEDLINE was searched with the following search string: (pulses[MeSH Terms] OR legumes[MeSH Terms] OR “food groups”) AND (cancer OR Neoplasms[MeSH Terms] OR obesity[MeSH Terms] OR dementia[MeSH Terms]) AND (meta-analysis[filter] OR systematic review[filter]). The search identified 101 hits that were screened, and the most recent and comprehensive systematic reviews for the above-mentioned outcomes are presented below. In this search, one umbrella review and systematic review and meta-analysis of prospective cohorts commissioned by European Association for the Study of Diabetes (EASD) published in 2019 was found (12). The searches for the *de novo* systematic review included articles indexed until the 16th of May 2022 and, correspondingly, our additional search included meta-analyses until the 24th of September 2022. The quality of the included articles in the systematic reviews used in the systematic search was assessed with the AMSTAR 2 tool with an NNR2023 adaption (13, 14), with the qualities categorised into high/moderate/low/very low. The *de novo* systematic review examined strength of evidence according to the World Cancer Research Fund criteria (15). Studies covered by other meta-analyses and systematic reviews and thus not selected are listed in Supplementary Table 1.

## Dietary intake in the Nordic and Baltic countries

In the Nordic and Baltic countries, the intake of legumes is generally relatively low, with mean consumption among adults ranging from 1 to 3 g/day in Denmark and Norway to 17–18 g in Latvia (16, 17). However, it is difficult to accurately assess the exact intake of legumes in dietary surveys because of daily variation in intake. Furthermore, foods from this food group could be included in various dishes and processed foods in amounts not always known to the consumer. Comparisons between countries may be further confounded due to differences in how legumes are classified.

## Health outcomes relevant for Nordic and Baltic countries

**Fig. 1:**
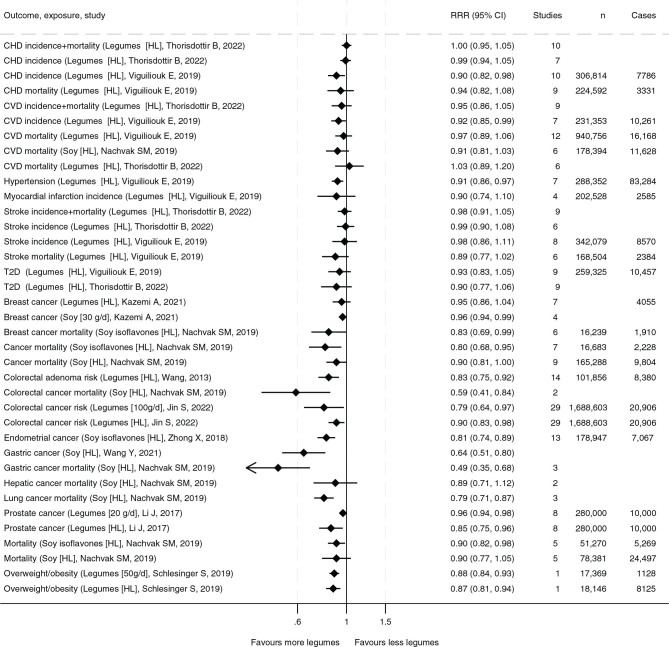
Summary of associations of relative risks ratios (RRR) from the most comprehensive meta-analyses between high compared to low (HL) consumption or dose-response (in specific grams) of legumes or soy, and morbidities and mortalities from cancer, cardiovascular disease, obesity, and type 2 diabetes (listed by first author & publication year). Number of studies, participants (n), and number of cases are also specified.

CVD and cancers are the two leading causes of death in the Nordic and Baltic countries (18, 19). As summarized in both the *de novo* systematic review for NNR2023 and the systematic review commissioned by EASD (3, 12), consumption of legumes is associated with longevity (reduced mortality), and legumes including soy are either inversely or neutrally associated with hypertension and coronary heart disease (CHD) (very low evidence, Table 1 & Fig. 1). Higher compared to lower consumption of legume and soy products such as tofu was generally associated with a slightly lower risk for CVD (Table 1). For several cancers and cancer-related mortalities, there were inverse associations with legume consumption including soy (mostly low evidence). There was no clear conclusion on type 2 diabetes. The details of these and other health outcomes will be presented below.

**Table 1:** Consumption of legumes and association to various health morbidities and mortalities from cardiovascular diseases, cancer, type 2 diabetes and obesity, and other outcomes from meta-analyses

First author	Year	Exposure	Outcome	Unit	Difference (95% CI)	Participants	Evidence	AMSTAR 2-NNR
Thorisdottir	2023	Legumes	CVD (incidence & mortality)	HL	RR 0.95 (0.86–1.06)	9 studies		High
Thorísdottír	2023	Legumes	CVD mortality	HL	RR 1.03 (0.89–1.20)	6 studies		High
Thorísdottír	2023	Legumes	CHD (incidence & mortality)	HL	RR 1.00 (0.95–1.05)	10 studies		High
Thorísdottír	2023	Legumes	CHD incidence	HL	RR 0.99 (0.94–1.05)	7 studies		High
Thorísdottír	2023	Legumes	Stroke (incid. & mortality)	HL	RR 0.98 (0.91–1.05)	9 studies		High
Thorísdottír	2023	Legumes	Stroke incidence	HL	RR 0.99 (0.91–1,09)	6 studies		High
Thorísdottír	2023	Legumes	T2D	HL	RR 0.90 (0.77–1.06)	9 studies		High
Thorísdottír	2023	Legumes	Total cholesterol	IvsC	−0.22 mmol/L (−0.32, −0.13)	14 studies		High
Thorísdottír	2023	Legumes	LDL-cholesterol	IvsC	−0.19 mmol/L (−0.27, −0.22)	12 studies		High
Thorísdottír	2023	Legumes	HDL-cholesterol	IvsC	ns	14 studies		High
Thorísdottír	2023	Legumes	Triglycerides	IvsC	ns	12 studies		High
Thorísdottír	2023	Legumes	Fasting glucose	IvsC	−0.19 mmol/L (−0.33, −0.5)	10 studies		High
Thorísdottír	2023	Legumes	Insulin	IvsC	ns	10 studies		High
Thorísdottír	2023	Legumes	HOMA-IR	IvsC	−0.30 (−0.60, 0.00)	7 studies		High
Viguiliouk	2019	Legumes	CVD incidence	HL	RR 0.92 (0.85–0.99)	7 studies, 231,353 participants, 10,261 cases	Low	High
Viguiliouk	2019	Legumes	CVD mortality	HL	RR 0.97 (0.89–1.06)	12 studies, 940,756 participants, and 16,168 cases.	Very low	High
Viguiliouk	2019	Legumes	CHD incidence	HL	RR 0.90 (0.83–0.99)	10 studies, 306,814 unique participants, and 7786 cases	Very low	High
Viguiliouk	2019	Legumes	CHD mortality	HL	RR 0.94 (0.82–1.08)	9 studies, 224,592 participants, and 3,331 cases.	Very low	High
Viguiliouk	2019	Legumes	Myocardial infarction incidence	HL	RR 0.90 (0.74–1.10)	4 studies, 202,528 participants, and 2,585 cases	Very low	High
Viguiliouk	2019	Legumes	Stroke incidence	HL	RR 0.98 (0.86–1.11)	8 studies, 342,079 participants, and 8,570 cases.	Very low	High
Viguiliouk	2019	Legumes	Stroke mortality	HL	RR 0.89 (0.78–1.03)	6 studies, 168,504 participants, and 2,384 cases	Very low	High
Viguiliouk	2019	Legumes	T2D	HL	RR 0.93 (0.83–1.05)	9 studies, 259,325 participants, and 10,457 cases.	Very low	High
Viguiliouk	2019	Legumes	Hypertension	HL	RR 0.91 (0.86–0.97)	7 studies, 288,352 participants, and 83,284 cases	Very low	High
Schlesinger	2019	Legumes	Overweight/ obesity	HL	RR 0.87 (0.81–0.94)	1 study, 18,146 participants, 1,825 cases	Very low	High
Schlesinger	2019	Legumes	Weight gain	50 g/day	RR 0.88 (0.84–0.93)	1 study, 18,146 participants	Very low	High
Schwingshackl	2017	Legumes	Mortality	HL	RR 0.96 (0.93–1.00)	17 studies, 53,085 cases	Moderate	High
Schwingshackl	2017	Legumes	Mortality	50 g/day	RR 0.96 (0.90–1.01)	17 studies, 53,085 cases	Moderate	High
Kazemi	2021	Legumes	Breast cancer	HL	RR 0.95 (0.87–1.05)	4 studies	Low	High
Kazemi	2021	Soy	Breast cancer	30 g/day	RR 0.96 (0.94–0.99)	7 studies, 4,055 cases	Moderate	High
Kim	2016	Legumes	Body weight	132 g/day	−0.34 kg (−0.63, −0.04)	21 trials, 940 participants	Low	High
Wang	2021	Soy	Gastric cancer	HL	RR 0.64 (0.51–0.80)	13 studies		Moderate
Zhong	2018	Soy isoflavones	Endometrial cancer	HL	OR 0.81 (0.74–0.89)	13 studies, 178,947 participants, 7,067 cases	Low	Moderate
Li	2017	Legumes	Prostate cancer	HL	RR 0.85 (0.75–0.96)	8 studies, 281,034 participants, 10,234 cases	Low	Low
Li	2017	Legumes	Prostate cancer	20 g/day	−3.7% (95% CI: −5.8%, −1.5%)	8 studies, 281,034 participants, 10,234 cases	Low	Low
Nachvak	2019	Soy isoflavones	Mortality	HL	RR 0.90 (0.82–0.98)	5 studies, 51,270 participants, 5,269 cases		Moderate
Nachvak	2019	Soy	Mortality	HL	RR 0.90 (0.77–1.04)	5 studies, 78,381 participants and 24,497 deaths		Moderate
Nachvak	2019	Soy	CVD mortality	HL	RR 0.91 (0.81–1.03)	6 studies, 178,394 participants, 11,628 deaths		Moderate
Nachvak	2019	Soy	Cancer mortality	HL	RR 0.90 (0.81–1.00)	9 studies, 165,288 participants, 9,804 deaths		Moderate
Nachvak	2019	Soy isoflavones	Cancer mortality	HL	RR 0.80 (0.67–0.94)	7 studies, 16,683 participants, 2,228 deaths		Moderate
Nachvak	2019	Soy isoflavones	Breast cancer mortality	HL	RR 0.83 (0.69–0.99)	6 studies, 16,239 participants and 1,910 deaths		Moderate
Nachvak	2019	Soy	Gastric cancer mortality		RR 0.49 (0.35–0.68)	3 studies		Moderate
Nachvak	2019	Soy	Lung cancer mortality		RR 0.79 (0.71–0.87)	3 studies		Moderate
Nachvak	2019	Soy	Colorectal cancer mortality		RR 0.59 (0.41–0.84)	2 studies		Moderate
Nachvak	2019	Soy	Hepatic cancer mortality		RR 0.89 (0.71–1.12)	2 studies		Moderate
Jin	2022	Legumes	Colorectal cancer risk	HL	RR 0.90 (0.83–0.98)	29 studies, 1,688,603 participants, 20,906 cases	Low	Low
Jin	2022	Legumes	Colorectal cancer risk	100 g/day	RR 0.79 (0.64–0.97)	29 studies, 1,688,603 participants, 20,906 cases	Low	Low
Wang	2013	Legumes	Colorectal adenoma risk	HL	RR 0.83 (0.75–0.93)	14 studies, 101,856 participants, and 8,380 cases		Low

The consumption comparison is listed as high versus low consumption (HL), intervention versus control group (I vs. C), or specific intake level.

### Cardiovascular disease

Meta-analyses of prospective cohort studies in the *de novo* systematic review on either total CVD, CHD, or stroke showed no associations comparing higher with lower legume consumption and no dose-response relationship (Table 1 & Fig. 1). The *de novo* systematic review did not include consumed soy products. The authors further cautioned that the result needs to be interpreted in the light of the generally low legume consumption reported in the cohorts. Randomized controlled trials on approximately >120–150 g/day of legumes indicate biological effects on biomarkers for disease, with these meta-analyses showing lowering of total cholesterol (−0.22 mmol/L (95% confidence interval [CI]: −0.32, −0.13) and LDL-cholesterol (−0.19 mmol/L, 95% CI: −0.27, −0.11), but no significant effect on triglycerides or high-density lipoprotein (HDL-C). Overall, the findings from the systematic review were mixed, with high heterogeneity and mostly moderate/some concern for risk of bias. The authors considered the evidence strong in supporting the absences of any adverse effects of legume intake in relation to CVD or type 2 diabetes. Furthermore, since legume interventions were suggested to have protective effects on blood lipids, the conclusion of the *de novo* systematic review was that the evidence was inconclusive on the causal relationship. The evidence for associations between legume consumption and CVD was judged as limited – no conclusion.

The EASD commissioned systematic review found that the highest compared with the lowest level of consumption of legumes including soy products, was associated with significant decreases in CVD (RR: 0.92; 95% CI: 0.85, 0.99), CHD (RR: 0.90; 95% CI: 0.83, 0.99) and hypertension (RR: 0.91; 95% CI: 0.86, 0.97) incidence (12). There was no association with myocardial infarction or stroke, or CVD, CHD, and stroke mortality. The lowest quantile of intake of dietary pulses with or without other legumes ranged from a median of 0 g/day for myocardial infarction incidence to 16.2 g/day for obesity incidence. The highest quantile of intake of dietary pulses with or without other legumes ranged from a median of 27.8 g/day for CVD mortality to 213 g/day for myocardial infarction incidence. The overall certainty of the evidence was graded as ‘low’ for CVD incidence and ‘very low’ for all other outcomes. The authors concluded that current evidence shows that legumes are associated with reduced CVD incidence with low certainty and reduced CHD and hypertension. Contrary to the *de novo* systematic review, the EASD systematic review included both peanuts and soybean products (e.g. tofu), which might explain the discrepancy in the findings (Tables 1–2 & Fig. 1).

**Table 2:** Summary of relevant findings regarding legumes and health from non-meta-analysis systematic reviews/umbrella reviews

First author	Year	Exposure	Outcome	Summary of results
Li N	2020	Soy isoflavones	All health outcomes	Umbrella review including 114 meta-analyses and systematic reviews. Generally, soy and isoflavone consumption is more beneficial than harmful. Beneficial associations were identified for cancers, cardiovascular disease, gynaecological, metabolic, musculoskeletal, endocrine, neurological, and renal outcomes.
Martini	2021	Legumes	All health outcomes	Umbrella review including six meta-analyses of observational studies. Concludes with possible association with decreased risk of colorectal cancer and CHD,
Messina	2022	Isoflavones and soy	Hormonal effects	After extensive review, the evidence does not support classifying isoflavones as endocrine disruptors.
Rafie	2017	Legumes	Telomere length	Two studies showed positive (beneficial) associations to telomere length and legume consumption while six showed neutral associations
Solfrizzi	2017	Legumes	Cognitive performance	Consumption of legumes associated with better cognitive performance (and cortical thickness)

### Cancers

A high consumption of legumes including soy is inversely associated with cancer mortality and risk of several cancers (20,21,22,23,24,25,26,27) (evidence mostly weak). A high compared to low consumption of legumes is associated with a relative risk (RR) of 0.90 (95% CI: 0.83–0.98). Correspondingly, for an increase in consumption of legumes of 100 g/day, RR is 0.79 (0.64–0.97). A high consumption of soy products is associated with a slightly lower risk of breast cancer. A high consumption of soy is also inversely associated with gastric, lung, and endometrial cancers (RR 0.64 [0.51–0.80], RR 0.79 [0.71–0.87], and OR 0.81 [0.74–0.89], respectively). High consumption of legumes is inversely associated with prostate cancer (RR 0.85 [0.75–0.96]).

### Type 2 diabetes

Type 2 diabetes and metabolic risk factors strongly contribute to life years lost in the Nordic and Baltic countries (18, 19). For type 2 diabetes, no association was found when comparing high and low intake of legumes in observational cohort studies in neither the *de novo* systematic review nor the EASF systematic review (3, 12).

Meta-analyses of randomized controlled trials with interventions increasing the intake of legumes found a significant decreased fasting glucose (−0.19 mmol/L, 95% CI: −0.33, −0.05) and insulin resistance (homeostatic model assessment for insulin resistance, −0.30, 95% CI: −0.60,−0.00) (15). The *de novo* SR concluded that the overall evidence for associations between consumption of legumes and risk of type 2 diabetes was considered as limited – no conclusion.

### Mortality and other morbidity

A high consumption of legumes is associated with decreased mortality (RR 0.96, 0.93–1.00, evidence rated as moderate) (28). For an increase in legume consumption of 50 g/day, the corresponding RR of mortality was 0.96 (0.90–1.01). A high consumption of soy isoflavones/soy products might also be associated with a reduction in mortality (RR 0.90 (0.82–0.98) / RR 0.90 [0.77–1.04]) (27).

Related to other morbidities, a high consumption of legumes is associated with less overweight and obesity (29) (RR 0.87 [0.81–0.94], evidence very low). Further, a high consumption of legumes has been shown to be associated with better cognitive performance (30).

## Mechanisms

Legumes generally contain high proportion of proteins and fibres, and some fatty acids with a composition associated with a reduction in CVD (31). They also contain several active compounds including flavonols and isoflavonols that are assumed to have anti-proliferative properties, which can explain some of the associations with cancers (27). Further, the World Cancer Research Fund has assessed the evidence that fibres are protective for colorectal cancers as strong (32). Generally, soy and isoflavone consumption is considered as more beneficial than harmful (5, 33). Further, some studies have also suggested a positive (beneficial) association between legume consumption and telomere length (34). Studies on pre-cancerous development such as colon adenomas also show similar associations as colorectal cancers (21).

A meta-analysis of trials of diets rich in legumes and the association with weight (35) mirrored the meta-analysis on overweight and obesity (29), showing significant weight reduction of −0.34 kg (95% CI: −0.63, −0.04 kg) with a consumption of 134 grams per day over 6 weeks compared to no intake.

There are also favourable associations between legume consumption and biomarkers of CVD and type 2 diabetes, including low density lipoproteins, total cholesterol, fasting glucose, and insulin sensitivity (3, 12). A systematic review assessing various biomarkers important for CVD and type 2 diabetes and ranking 10 food groups from most to least beneficial for these biomarkers, ranked legumes as second in terms of most beneficial biomarker profile behind nuts and before grains, fruits and vegetables, eggs, dairy, and fish, with red meat, and sugar-sweetened beverages on the least beneficial part of the spectrum (36). Prospective cohort studies have observed inverse associations between consumption of legumes and hypertension (12).

## Food-based dietary guidelines

A high consumption of legumes, and soy in particular, is associated with a decreased risk of mortality from gastric, colorectal, breast, endometrial, and lung cancers (20,21,22,23,24,25,26,27). Inclusion of legume and soy products such as tofu, is associated with favourable biomarkers of CVD and type 2 diabetes (3, 12). They seem to be associated with a reduction in all-cause mortality and potentially CHD (3, 12, 27), although the evidence is very low, and when excluding soy products, the association between legumes and CVD is neutral (15). However, considering both the favourable changes in biomarkers together with the associations to CVD and type 2 when including soy products (3, 12), there seems to be some beneficial effects of legumes related to cardiovascular health. Legume consumption is also associated with less obesity (29); the inclusion of legumes in the diet may be beneficial for people aiming for weight loss even when diets are not intended to be calorically restricted (35). One concern has been related to hormonal effects of soy products. However, an extensive review of potential endocrine disruption, does not support such concerns (37). A risk assessment of intake of phytoestrogens found in soy products such as genistein concluded that there was no risk for exceeding a health-based guidance upper intake value in pregnant women, but that children replacing animal products with primarily soy products including soy drinks could exceed the guidance value for genistein (5). Legumes might contribute beneficially also to cognitive outcomes (30).

Allergies and related adverse reactions to most legumes are relatively uncommon (38). Reactions to soy are not uncommon, but these reactions are rarely severe. With proper food labelling and storage of food, such allergies and related reactions could be limited (38). Also, lectins found in many varieties of pulses could cause unfavourable health effects, such as nausea, vomiting, bowel pain, and diarrhoea, if beans and peas are improperly cooked or are consumed uncooked. Dried beans and peas should be soaked in water overnight before rinsed, and boiled until soft (39).

## Integration

The current evidence supports legume consumption as part of healthy diets to reduce the risk of later chronic disease. Based on consumption and morbidity data in the Nordic and Baltic countries and the data on mortality, increasing the consumption of legumes might contribute to some increase in life expectancy. Sustained change in the consumption of legumes from none to 100 grams per day is associated with decreased mortality (28), with an increase in life expectancy of approximately 1 year for male and female adults in the age range 30 to 50 years (40). Legumes are further among the most sustainable diet components (13).

The current evidence has some shortcomings. Several studies have assessed sub-group analyses for soy, but few have presented specific estimates for morbidities related to consumption of other specific groups of legumes. Legume consumption in populations participating in cohort studies is in general low, making it difficult to assess health effects of high intakes. Also, for children, little evidence is available.

Overall, the current evidence supports a dietary recommendation to increase legume consumption.

## Conflict of interest and funding

The authors have not received any funding or benefits from industry to conduct this study, and report no conflicts of interests. The authors received a small reimbursement from the Norwegian Directorate of Health for work linked to this article.

## Supplementary Material


