# Supplementary material for: Legumes and pulses - a scoping review for Nordic Nutrition Recommendations 2023
Source: Food Nutr Res. 2024 Mar 26;68:10.29219/fnr.v68.10484. doi: 10.29219/fnr.v68.10484 (PMC10989235; doi:10.29219/fnr.v68.10484)
Supplement: Supplementary file 1 [file FNR-68-10484-s1.pdf]

Supplementary material

Supplementary Table 1: Studies not selected as they are covered by other meta-analyses and systematic reviews.

First author	Year of publication	Exposure	Outcome	Covered by
Lu	2017	Soy	Gastrointestinal	Jin et al.
Marventano	2017	Legumes	CVD	Thorísdottír and Viguiliouk et al.
Zhu	2015	Legumes	Colorectal cancer risk	Jin et al.
Namazi	2018	Soy	Mortality	Viguiliouk et al.
Pearce	2021	Legumes	Diabetes type 2	Thorísdottír and Viguiliouk et al.
Qin	2019	Isoflavones and soy	Fibrinoids	Messina et al.
Schwingshackl	2018	Legumes	Colorectal cancer risk	Jin et al.
Viguiliouk E	2017	Legumes	CVD	Thorísdottír and Viguiliouk et al.
Yang W	2011	Soy	Lung cancer	Nachvak et al.
Yu Y	2016	Soy isoflavone	Colorectal cancer risk	Nachvak et al.
Zheng	2016	Soy isoflavone	Osteoporosis	Messina et al.

* Abbreviations: CVD: cardiovascular disease
